# Face‐Fusion of Icosahedral Boron Hydride Increases Affinity to γ‐Cyclodextrin: *closo*,*closo*‐[B_21_H_18_]^−^ as an Anion with Very Low Free Energy of Dehydration

**DOI:** 10.1002/cphc.201901225

**Published:** 2020-04-07

**Authors:** Khaleel I. Assaf, Josef Holub, Eduard Bernhardt, Josep M. Oliva‐Enrich, M. Isabel Fernández Pérez, Moisés Canle, J. Arturo Santaballa, Jindřich Fanfrlík, Drahomír Hnyk, Werner M. Nau

**Affiliations:** ^1^ Department of Life Sciences and Chemistry Jacobs University Bremen Campus Ring 1 28759 Bremen Germany; ^2^ Department of Chemistry Al-Balqa Applied University 19117 Al-Salt Jordan; ^3^ Institute of Inorganic Chemistry of the Czech Academy of Sciences 25068 Husinec-Řež Czech Republic; ^4^ Bergische University Wuppertal Gaussstrasse 20 42097 Wuppertal Germany; ^5^ Instituto de Química-Física “Rocasolano” CSIC, ESP- 28006 Madrid Spain; ^6^ Departamento de Química Facultade de Ciencias and CICA Zapateira Universidade da Coruña Grupo de Reactividade Química e Fotorreactividade (REACT!) ESP- 15071 Coruña Spain; ^7^ Institute of Organic Chemistry and Biochemistry of the Czech Academy of Sciences Flemingovo nam. 2 16610 Prague Czech Republic

**Keywords:** anion binding, boron clusters, desolvation, host-guest chemistry, intermolecular interactions

## Abstract

The supramolecular recognition of *closo,closo*‐[B_21_H_18_]^−^ by cyclodextrins (CDs) has been studied in aqueous solution by isothermal titration calorimetry and nuclear magnetic resonance spectroscopy. These solution studies follow up on previous mass‐spectrometric measurements and computations, which indicated the formation and stability of CD ⋅ B_21_H_18_^−^ complexes in the gas phase. The thermodynamic signature of solution‐phase binding is exceptional, the association constant for the γ‐CD complex with B_21_H_18_^−^ reaches 1.8×10^6^ M^−1^, which is on the same order of magnitude as the so far highest observed value for the complex between γ‐CD and a metallacarborane. The nature of the intermolecular interaction is also examined by quantum‐mechanical computational protocols. These suggest that the desolvation penalty, which is particularly low for the B_21_H_18_^−^ anion, is the decisive factor for its high binding strength. The results further suggest that the elliptical macropolyhedral boron hydride is another example of a CD binder, whose extraordinary binding affinity is driven by the chaotropic effect, which describes the intrinsic affinity of large polarizable and weakly solvated chaotropic anions to hydrophobic cavities and surfaces in aqueous solution.

## Graphical abstract

**High affinity**! The [B_21_H_18_]^−^ ion is a new example of a superchaotropic anion that forms a stable host–guest complex with cyclodextrins in aqueous solution.

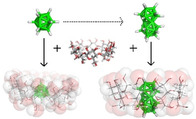

Boron cluster chemistry is dominated by icosahedrally shaped cages (Figure 1), which can be exemplified by *closo*‐B_12_H_12_^2−^. Larger clusters can be obtained formally by their mutual fusion. The *closo,closo*‐[B_21_H_18_]^−^ ion, **B21**, is an example of shared icosahedral moieties with three joint vertices.^[1]^ The COSAN ion (CObalt SANdwich, Co(C_2_B_9_H_11_)_2_^−^) represents another way of fusion, i. e., via a single vertex.^[2]^ **B21** exhibits a low chemical reactivity, which resists most reaction conditions except for fluorination.^[3]^ However, complexation of the potassium salt of **B21** with β‐ and γ‐cyclodextrin (CDs, naturally occurring macrocycles that are water‐soluble and formed by oligomerization of seven or eight glucopyranoside units, respectively)^4^, ^5^, ^6^ has already been observed in the gas phase.^7^ Quantum‐chemical computations in the gas phase suggested dihydrogen bonds between the B−H vertices and the polar hydroxyl groups of CDs.^7^ Since the interaction of boron clusters with CDs in the aqueous solution phase might open novel avenues towards interactions with other water‐soluble biomolecules, we now extended our studies of **B21** to the solution phase.^8^, ^9^

**Figure 1:**
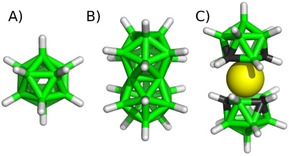
Molecular structures of A) B_12_H_12_^2−^, B) B_21_H_18_^−^, and C) *meta*‐COSAN, Co(C_2_B_9_H_11_)_2_^−^. Color coding is as follows: boron‐green, carbon‐black, hydrogen‐white, cobalt‐yellow.

High‐affinity (micromolar) binding in aqueous solution has recently been documented between various COSANs and CDs.^10^ However, the *closo*‐B_12_H_12_^2−^ cluster, the smaller building block of **B21**, only exhibits a lower affinity (millimolar) to γ‐CD.^11^ The association constant was considerably increased by halogenation, up to an affinity of 10^6^ M^−1^ for B_12_Br_12_^2−^ with γ‐CD.^11^ The complexation of *closo*‐B_12_X_12_^2−^ to the hydrophobic inner cavity of CDs appears counterintuitive at first glance, due to the doubly negative charge of these clusters and their high water solubility (hydrophilicity). The high affinity of such large anionic clusters, not only borate clusters but also others such as polyoxometalates^12^, ^13^, ^14^, ^15^, ^16^ or octahedral metal clusters,^13^, ^17^ to hydrophobic cavities as well as to neutral surfaces, membranes, and proteins has been described as the chaotropic effect^11^, ^18^, ^19^ that presents a topic of considerable current interest. Accordingly, the propensity of anionic clusters to associate to hydrophobic cavities has been attributed to (*i*) the comparative ease of desolvation of the large clusters in combination with (*ii*) the stabilization of the resulting assemblies by dispersion interactions, promoted by the high polarizability of these large anions.

In this study, we carried out an experimental and computational investigation of the complex formation between **B21** and CDs in aqueous solution in order to get a deeper insight to binding of icosahedron‐based elliptic boron clusters and compare them with their closest relatives, B_12_X_12_^2−^ and COSANs, see Figure 1. Through this comparison, we tried to advance structure‐affinity relationships in the emerging field of chaotropic anion recognition.

We prepared **B21** according to literature procedures.^1^, ^7^ The formation of host‐guest complexes between **B21** (as sodium salt) and CDs was first studied by ^1^H NMR spectroscopy. No significant changes in the ^1^H NMR spectra were observed with α‐CD, which is expected due to its small cavity size. In contrast, large complexation‐induced chemical shifts were obtained with β‐CD and γ‐CD, see Figure 2, which indicated the formation of host‐guest complexes in aqueous solution, qualitatively in agreement with the gas‐phase results.^7^ In particular, we observed pronounced complexation‐induced chemical shifts for the H3 and H5 protons, which are located inside the cavity, signaling the formation of deep inclusion complexes, in analogy to the complexation of perhalogenated *closo*‐B_12_X_12_^2−^ anions with CDs.^11^, ^20^ The fact that the two diasterotopic H6 protons of the larger CDs split into an AB system upon complexation can be accounted for by a hindered rotation of the CH_2_OH groups upon complexation. The larger shifts obtained upon complexation of **B21** with the largest investigated macrocycle, γ‐CD, indicated the formation of a tightly packed complex. The potassium salt of **B21** provided identical NMR results, which demonstrated that the counter‐ion plays no major role in the complexation in water. In contrast to the large ^1^H NMR shifts of the H3 and H5 CD protons, the ^11^B chemical shifts^21^ of **B21** in the complex showed comparably less diagnostic changes with respect to uncomplexed **B21**, see Figure S1 in the Supporting Information.

**Figure 2:**
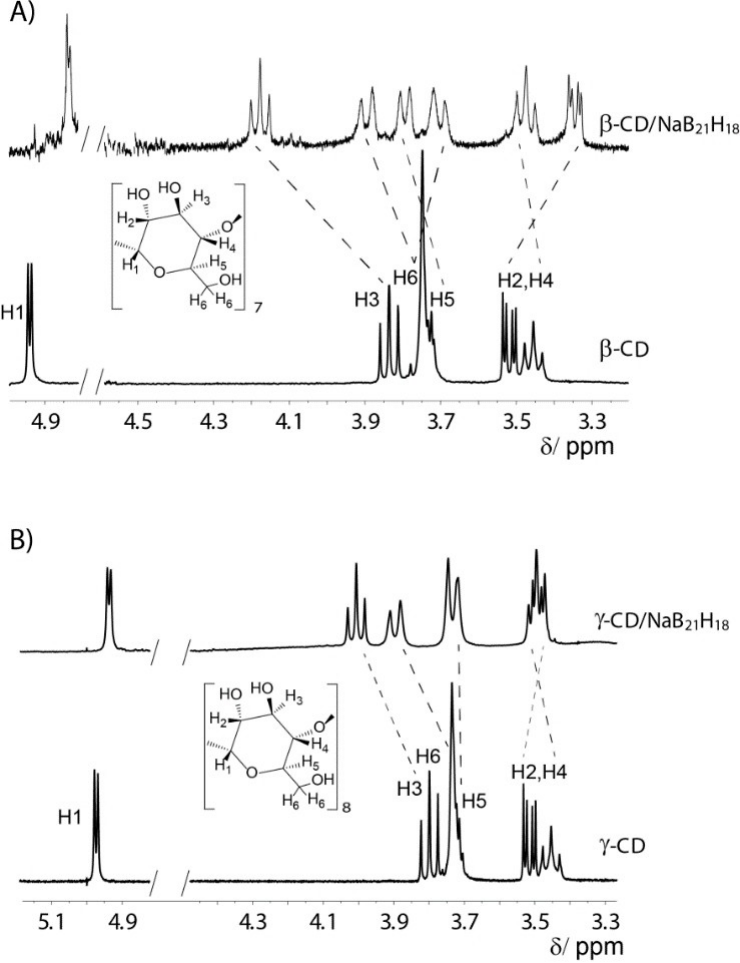
^1^H NMR spectra of A) free β‐CD and the β‐CD/NaB_21_H_18_ complex and B) free γ‐CD and the γ‐CD/NaB_21_H_18_ complex, both measured in D_2_O.

Isothermal titration calorimetry (ITC) was used to determine the absolute binding constants and to analyze the complexation thermochemistry (Figure 3); access to this technique was essential, because the binding turned out to be too strong to employ NMR titrations for accurate determination of the binding constants (*K*_a_). An ITC dilution titration of **B21** (1 mM) was performed first; no significant heat effect was observed, which excluded a competitive enthalpic effect due to micellization/de‐aggregation events (reported for *ortho*‐COSANs),^22^, ^23^, ^24^ and the subsequent host‐guest titrations were performed at much lower **B21** concentrations (0.1 mM). The ITC titration profiles were consistent with a 1 : 1 binding stoichiometry, consistent with the mass‐spectrometric study, in which also no 2 : 1 host‐guest complexes had been observed.^7^ The *K*_a_ values obtained by ITC for the complexation of **B21** (as sodium salt) with β‐CD and γ‐CD were found to be (1.3±0.1) ×10^5^ and (1.8±0.5) ×10^6^ M^−1^, respectively. These values are on the same order of magnitude as those measured for *meta*‐COSAN,^10^ twice higher than those obtained for the most tightly binding single icosahedron, i. e., B_12_Br_12_,^2−[11]^ and they significantly exceed the affinities of other “mononuclear” borate clusters or carboranes.^25^ The same trend applies for the B_12_H_12_^2−^ cluster, whose association constant with γ‐CD is even smaller, by three orders of magnitude.^11^ The ITC data showed that the complexation is enthalpically driven. The large Δ*H* values are compensated by large entropic penalties, which are jointly in line with the thermochemical fingerprint of the chaotropic effect.^11^, ^13^, ^15^, ^16^, ^17^, ^19^, ^20^, ^25^, ^26^

**Figure 3:**
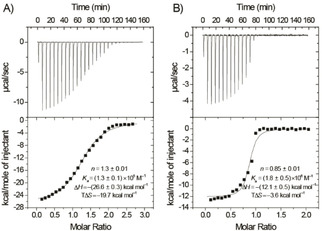
Microcalorimetric titrations of B_21_H_18_^−^ (as sodium salt) with A) β‐CD and B) γ‐CD. Top: Raw ITC data for sequential twenty‐seven injections of host solution (1.0 mM) into the guest solution (0.1 mM). Bottom: Apparent reaction heats obtained by integration of the calorimetric traces and fitting for a 1 : 1 complexation model. The deviations from the ideal value for the stoichiometric parameter *n* are attributed to uncertainties in absolute concentrations due to water and salt content. Note that a common concentration determination, e. g., with a ^1^H NMR reference standard, is not feasible for the hybrid organic‐inorganic host‐guest system.

We also used a computational model to gain deeper insight into the binding of **B21** to γ‐CD and expanded this investigation, for comparison, to other compounds with icosahedral γ‐CD binding motifs, specifically *ortho*‐COSAN, *meta*‐COSAN (*ortho* and *meta* define the positions of the respective C atoms in the COSAN framework),^10^ B_12_H_11_NH_3_^−^, B_12_H_12_^2−^, B_12_Cl_12_^2−^, B_12_Br_12_^2−^, and B_12_I_12_^2−^. For all these compounds, association constants (*K*_a_) are known under the same conditions (as sodium salts, in H_2_O) and a predominant 1 : 1 binding stoichiometry can be assumed.^11^, ^20^

Very important to consider, boron clusters as well as other large anions have been found to act as so‐called superchaotropic anions, that is, they display a special hydration behavior,^11^, ^18^, ^19^ which can be classically described as a “water‐structure breaking” effect or in terms of a weak hydration. Accordingly, it was imperative to consider, in addition to gas‐phase interactions energies, the free energies of hydration of all involved species. The environment was described by using implicit solvent models (COSMO and SMD); only the sodium counterion was treated as a by‐stander in the host‐guest complexation process, in line with the observed absence of a counter‐ion effect when changing to potassium as cation. The known crystal structure of B_12_Br_12_^2−^ with γ‐CD was employed to build starting geometries for our quantum mechanics‐based molecular dynamic/quenching (MD/Q) simulations.^11^ The most favorable structures from the MD/Q simulations are shown in Figure 4, and the computed binding ‘free’ energies (Δ*G*′) are summarized in Table 1. Note that **B21** is almost fully immersed inside γ‐CD (Figure 4A), in line with the observed 1 : 1 complexation pattern. The Δ*G*′ values were decomposed into three terms, i. e., the gas‐phase interaction energy (Δ*E*), the change of solvation free energy upon complex formation (ΔΔ*G*_solv_), and the change of the conformational ‘free’ energy upon complex formation (Δ*G*′_conf_), see Ref. [27] for details.

**Figure 4:**
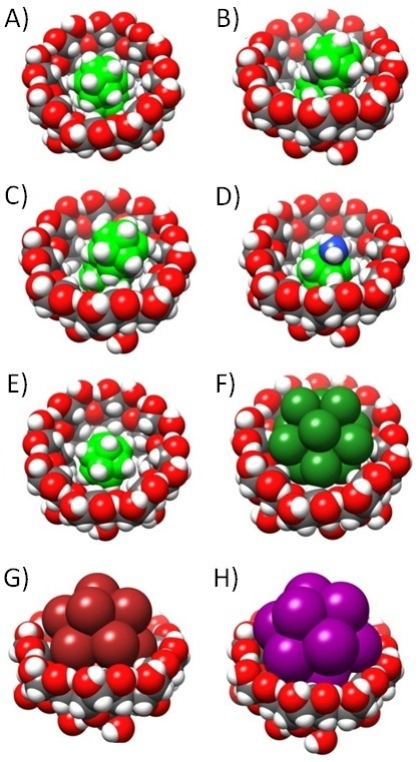
The most stable computed structures of the γ‐CD host‐guest inclusion complexes with A) **B21**, B) *meta*‐COSAN, C) *ortho*‐COSAN, D) B_12_H_11_NH_3_^−^, E) B_12_H_12_^2−^, F) B_12_Cl_12_^2−^, G) B_12_Br_12_^2−^, and H) B_12_I_12_^2−^.

**Table 1:** Experimental association constant (*K*_a_) of boron clusters with γ‐CD, polarizability (*α* in Å^3^), experimental binding free energy (Δ*G* ^0^), computed binding ‘free’ energy (Δ*G* ^’^), interaction energy (Δ*E*), change of hydration free energy upon complex formation (ΔΔ*G* ^hydr^), hydration free energies of the clusters [Δ*G* ^hydr^(cluster)) as well as of the γ‐CD ⋅ cluster complexes (Δ*G* ^hydr^(complex)], and conformational distortion ‘free’ energy (ΔG’_conf_). All energies given in kcal mol^−1^. The non‐tabulated hydration free energy of γ‐CD amounts to −69.2 kcal mol^−1^.

Cluster	*K*_a_ [10^3^ M^−1^]	*α* ^[a]^	Δ*G* ^0^	Δ*G*′	Δ*E*	ΔΔ*G* ^hydr^	Δ*G* ^hydr^(cluster)	Δ*G* ^hydr^(complex)	Δ*G*′_conf_
mono‐anions									
B_21_H_18_^−^ (**B21)**	1800±200	36.7	−8.5	−32.5	−42.7	9.2	−23.1	−83.1	1.0
*meta*‐COSAN^−^	3000^[b]^	39.3	−8.8	−28.9	−39.9	10.5	−24.6	−83.3	0.5
*ortho*‐COSAN^−^	191^[b]^	39.7	−7.2	−22.5	−40.1	17.2	−33.4	−85.4	0.3
B_12_H_11_NH_3_^−^	1.7^[c]^	24.5	−4.4	−9.5	−44.8	33.6	−63.7	−99.3	1.7

di‐anions									
B_12_H_12_^2−^	2.0^[c]^	25.7^[c]^	−4.5	−6.1	−75.6	67.0	−163.6	−165.8	2.6
B_12_Cl_12_^2−^	17^[c]^	46.7^[c]^	−5.8	−15.7	−58.9	41.9	−127.6	−154.9	1.3
B_12_Br_12_^2−^	960^[c]^	58.2^[c]^	−8.2	−27.1	−60.6	32.2	−116.8	−153.8	1.3
B_12_I_12_^2−^	67^[c]^	84.1^[c]^	−6.6	−15.1	−62.3	44.2	−127.3	−152.3	3.0

[a] Calculated in the gas phase by using the B3LYP/aug‐cc‐pvdz method in the Gaussian 09 software. [b] Taken from ref. [10]. [c] Taken from ref. [11].

Expectedly, there is no absolute agreement between the experimental and calculated data (columns with Δ*G*_0_ and Δ*G*′ in Table 1). Among other reasons, the COSMO continuum solvation model has not been optimized for macromolecular species with concave interiors. Secondly, although high‐energy cavity water contributes comparably less to the driving force of host‐guest complexation for large cavities (such as that of γ‐CD) than for smaller ones,^28^ an energetic offset will nevertheless result from this neglect. Third, the SMD continuum solvation model (employed for the free borate clusters) has not been optimized for large polarizable ions and does not incorporate boron‐specific coordinative bonding effects of the H_2_O−H−B type, which have been previously considered in the hydration of borate clusters.^29^

Nevertheless, regardless of the absolute variation, the Δ*G*′ values show a very good linear correlation with the experimentally determined binding free energies (Δ*G* ^0^ values, *R* ^2^=0.94, *n*=8, see Chart 1), which demonstrates that the computational model picks up essential contributions to the driving force of the host‐guest complexation process. Besides the correctly reproduced overall trend, the model predicts salient experimental details, namely (*i*) the strongest binding (most negative Δ*G*′ values) for **B21** and *meta*‐COSAN, (*ii*) the significantly stronger binding of *meta*‐COSAN than *ortho*‐COSAN,^10^ and (*iii*) the maximal binding for X=Br in the B_12_X_12_^2−^ series. 
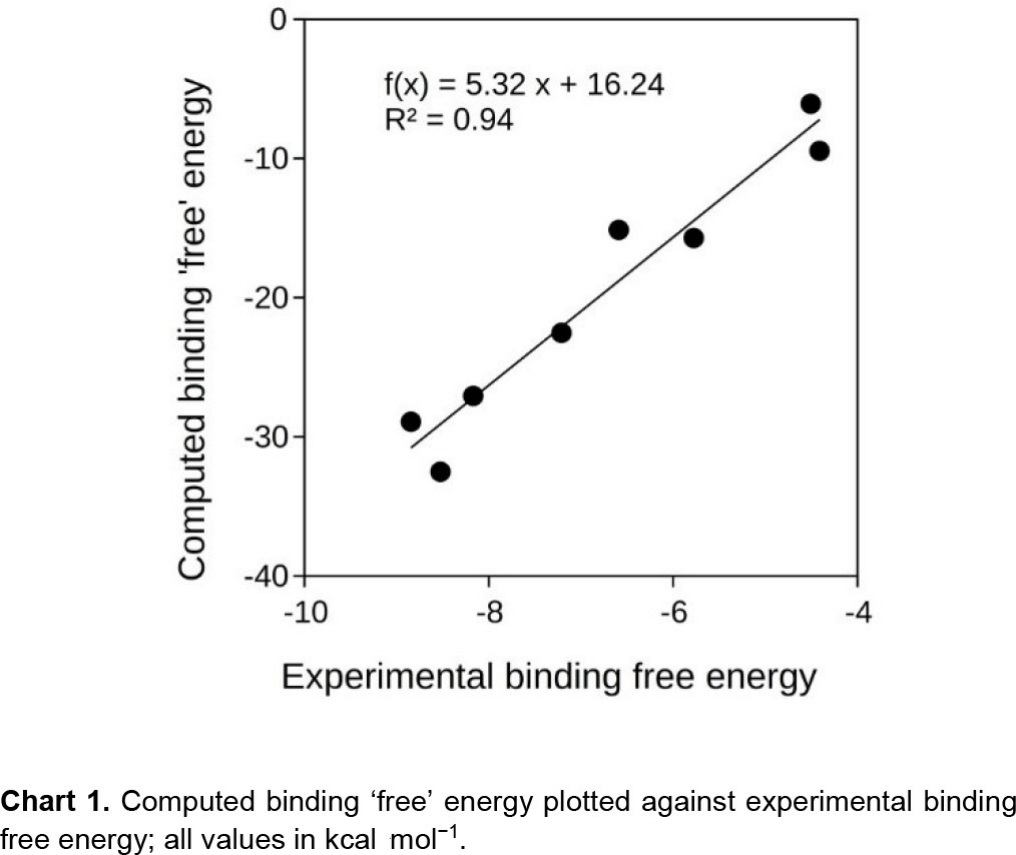


In searching (*i*) for the driving forces responsible for the high affinities and (*ii*) for the intermolecular interactions underlying the observed correlation, we could disregard three factors under discussion. First, *host* desolvation effects, related to emptying the cavity of γ‐CD upon guest binding, should be comparable along the series; they can be presumed to remain constant and cannot account for the large variations with cluster type. Second, the conformational free energies upon complex formation (Δ*G*′_conf_) are quite small (0.3 to 3.0 kcal mol^−1^) and cannot account for the much larger variations in Δ*G*′.^30^ Third, dihydrogen bonding does not dominate the host‐guest complexation process in water. The gas‐phase geometry optimizations of the host‐guest complexes do, indeed, predict the formation of numerous dihydrogen bonds between the protiated clusters and γ‐CD, in which the partially negative H atoms of the borate clusters interact with the partially positive H3 and H5 hydrogens of γ‐CD, see Figure 5.^31^ However, although such coordinative bonds may be very important in the gas‐phase^7^ and structure‐determining in the solid‐state,^32^, ^33^ they are not expected to present a sizable driving force in aqueous solution, because the same type of bonds will be formed with the protic O−H hydrogens of bulk water molecules, and these H_2_O−H−B bonds are likely stronger than those inside CDs. Indeed, despite the assigned dihydrogen bonds, **B21** and *meta*‐COSAN did not have highly negative Δ*E* values, which indicated that these dihydrogen bonds are not very strong, and are not the major driving force for complexation. It is worth noting that COSANs can exist as three rotamers (*cisoid*, *gauche*, and *transoid*) that differ in dipole moment, and among which the *cisoid* one is presumed to dominate in polar media.^10^, ^22^, ^34^, ^35^

**Figure 5:**
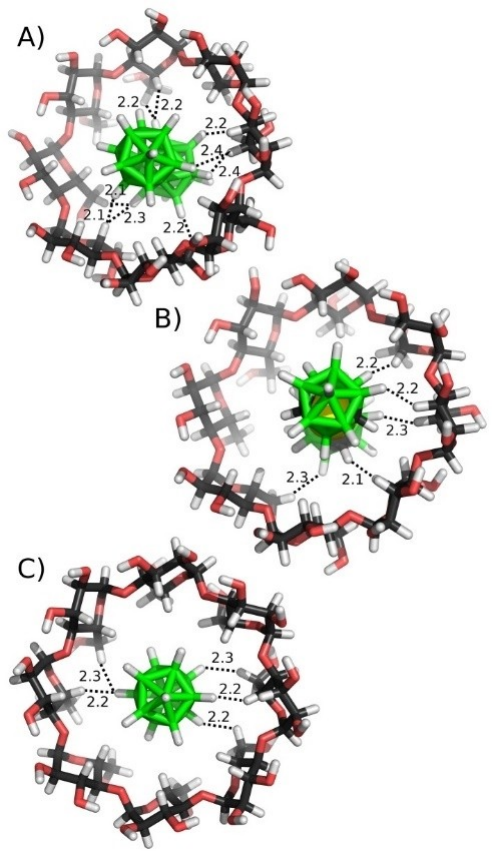
The most stable computed gas‐phase structures of γ‐CD with A) B_21_H_18_^−^ (**B21**), B) *meta*‐COSAN, and C) B_12_H_12_^2−^ ; B−H−H−C dihydrogen bonds <2.5 Å are indicated as dashed lines, with distances given in Å.

We therefore returned to the two more likely contributors, dispersion interactions, which should be governed by the polarizabilities of the clusters (see Table 1), and *guest* desolvation effects for the uncomplexed borate cluster. Desolvation effects should be absent in the gas phase, such that the Δ*E* values should mainly report on dispersions interactions as well as other bonding effects. Indeed, the equally large positional isomers *meta*‐COSAN and *ortho*‐COSAN do not differ significantly in polarizability (Table 1) and show the same Δ*E* values, within ±0.1 kcal mol^−1^. Moreover, the trend in Δ*E* values for B_12_I_12_^2−^>B_12_Br_12_^2−^>B_12_Cl_12_^2−^ coincides with the polarizability trend of these globular halogenated clusters (Table 1).^11^, ^19^ The Δ*E* values for B_12_H_12_^2−^ is very high (negative) despite its lower polarizability, which can be rationalized by the electrostatically driven dihydrogen bonding interactions in the gas phase; they do not contribute to the driving force in solution, because the same or stronger interactions apply in aqueous bulk (see above). However, although contributions arising from dispersion interactions must contribute, they do not overall correlate well with the experimental binding energies, such that our final analysis narrowed down on differential dehydration as main dominator to the driving force of host‐guest complexation, an effect which was previously postulated^11^, ^19^ but which has never been quantitatively inspected for large cluster anions up to now.

Towards this end, we further dissected the calculated differential desolvation term into the hydration free energies of the clusters and those of the γ‐CD ⋅ cluster complexes. For the associated discussion, we furthermore focused on two subsets, for which the hydration free energies of the γ‐CD ⋅ cluster complexes were very similar: the *meta*‐COSAN/**B21**/*ortho*‐COSAN mono‐anion subset and the B_12_Cl_12_^2−^, B_12_Br_12_^2−^, and B_12_I_12_^2−^ di‐anion subset. The reason why the hydration free energies are similar within the two subsets is due to the fact that the encapsulated clusters are shielded from bulk water, although there remains a sizable difference between the mono‐anion and di‐anions subsets as a consequence of the incomplete charge screening by the macrocycle. Within these subsets, the differential desolvation energies can be directly attributed to variations of the clusters themselves.

For the mono‐anion subset with *meta*‐COSAN, **B21**, and *ortho*‐COSAN, where Δ*G* ^hydr^(complex) is similar, within −83 to −85 kcal mol^−1^, the Δ*G* ^hydr^(cluster) values show a much larger spread from −23 to −33 kcal mol^−1^. Within this subset, the lower (less negative) calculated (and experimental) binding free energy of *ortho*‐COSAN can be traced back to its much larger (more negative) hydration free energy. *Ortho*‐COSAN (in its *cisoid* conformation) has a more than twice larger value of the dipole moment than *meta*‐COSAN (directions of these vectors are identical).^10^ *Ortho*‐COSAN is therefore better electrostatically hydrated than *meta*‐COSAN and the hydration shell is more difficult to strip off in the binding process, which leads to its weaker binding to γ‐CD. Since the dipole moment of the *D*_3h_‐symmetrical **B21** is zero on account of symmetry, we employed also the molecular electrostatic potential surfaces (MEPs) of **B21** and COSANs to pin‐point the source of the low desolvation of **B21**. Indeed, **B21** and *meta*‐COSAN have a lower magnitude of the MEP than *ortho*‐COSAN (−74.8, −79.1 and −87.7 kcal mol^−1^, respectively, see also Table S1).

The di‐anion subset includes B_12_I_12_^2−^, B_12_Br_12_^2−^, and B_12_Cl_12_^2−^, where Δ*G* ^hydr^(complex) is much larger but also similar, within −152 to −155 kcal mol^−1^, but where Δ*G* ^hydr^(cluster) shows a much more pronounced spread from −117 to −128 kcal mol^−1^. In this set of three, the brominated cluster B_12_Br_12_^2−^ stands out experimentally and theoretically, because it has the highest affinity but the lowest (least negative) hydration free energy, which – in relative terms – facilitates its binding to γ‐CD. The irregularity in the calculated hydration free energies (maximum for B_12_Br_12_^2−^) is likely due to the fact that the hydration of the largest cluster, B_12_I_12_^2−^, is disfavored due to its lower charge density but favored due to its very high polarizability. Note, in this context, that the polarizability of any hydrated species is an important determinant of its solubility, which accounts, for example, for the larger aqueous solubility of xenon than neon.^36^

To sum up, we reported an exceptional thermodynamic signature for the formation of the host‐guest inclusion complex between *closo,closo*‐[B_21_H_18_]^−^ (**B21**) and γ‐CD; the corresponding affinity reaches a micromolar value, 1.8×10^6^ M^−1^. Although the original quantum‐chemical analysis indicated that *closo,closo*‐[B_21_H_18_]^−^ interacts with γ‐CD *via* formation of numerous dihydrogen bonds in the gas phase, a detailed analysis in comparison with other boron clusters revealed that, in addition to dispersion interactions, the desolvation of the anionic clusters governs the trend in affinities of the different boron clusters with γ‐CD in aqueous solution. Accordingly, the exceptional affinity of **B21** to γ‐CD is enabled by a very small desolvation penalty, thereby qualifying macropolyhedral boron hydrides as new and impressive examples of superchaotropic anions.

## Experimental Section

Experimental and computational details are given in the Supporting Information.

## Conflict of interest

The authors declare no conflict of interest.

## Supporting information

As a service to our authors and readers, this journal provides supporting information supplied by the authors. Such materials are peer reviewed and may be re‐organized for online delivery, but are not copy‐edited or typeset. Technical support issues arising from supporting information (other than missing files) should be addressed to the authors.

Supplementary
