# Supplementary material for: Face‐Fusion of Icosahedral Boron Hydride Increases Affinity to γ‐Cyclodextrin: closo,closo‐[B21H18]− as an Anion with Very Low Free Energy of Dehydration
Source: Chemphyschem. 2020 Apr 7;21(10):971–6. doi: 10.1002/cphc.201901225 (PMC7318346; doi:10.1002/cphc.201901225)
Supplement: Supplementary file 1 — Supplementary [file CPHC-21-971-s001.pdf]

ChemPhysChem

Supporting Information

Face-Fusion of Icosahedral Boron Hydride Increases Affinity to γ-Cyclodextrin: *closo,closo*-$[B_{21}H_{18}]^{-}$ as an Anion with Very Low Free Energy of Dehydration

Khaleel I. Assaf,* Josef Holub, Eduard Bernhardt, Josep M. Oliva-Enrich, M. Isabel Fernández Pérez, Moisés Canle, J. Arturo Santaballa, Jindřich Fanfrlík,* Drahomír Hnyk,* and Werner M. Nau*© 2020 The Authors. Published by Wiley-VCH Verlag GmbH & Co. KGaA.
This is an open access article under the terms of the Creative Commons Attribution License, which permits use, distribution and reproduction in any medium, provided the original work is properly cited.

Table of Contents

1. Computed compound properties 2

2. Experimental section 3

2.1. NMR measurements 3

2.2. ITC measurements 3

2.3. Computational details 3

3. References 4

1. Computed compound properties

We computed the molecular electrostatic potential surfaces (MEP, computed on 0.001 isoelectron density) and molecular size parameters of the borate clusters, see Table S1. The latter characterize the elliptic *versus* globular structures in the two series of selected mon- and di-anions. As can be seen, the molecular dimensions of the toroidal cavity of γ-CD (radius of lower rim: 3.75 Å; radius of upper rim: 4.25 Å; cavity height: 8.6 Å) fit particularly nicely to the elliptical borate clusters, while the globular clusters have somewhat too large or too small radii, see optimized structures in Fig. 4 of the main text.

Table S1. Magnitude of molecular electrostatic potential surfaces ($V_{S,min}$) and molecular size parameters (radii r_1 and r_2). The sizes were computed from the maximal diameter of the molecule (r_1) and the maximal diameter within a single icosahedron (r_2) plus the van der Waals radii (1.20, 1.75, 1.83 and 1.98 Å for H, Cl, Br, and I, respectively). Energies are in kcal mol^{-1} and radii in Å. Molecular electrostatic potential surfaces were computed at the BLYP/def2-TZVP level.

Cluster	$V_{S,min}$	r_1	r_2
Elliptic mono-anions			
meta-$Co(C_2B_9H_{11})_2^-$	−79.1	6.37	4.11
$B_{21}H_{18}^-$ (**B21**)	−74.8	5.19	4.11
ortho-$Co(C_2B_9H_{11})_2^-$	−87.7	6.35	4.11
$B_{12}H_{11}NH_3^-$	−107.7	4.46	4.11
Spherical di-anions			
$B_{12}Br_{12}^{2-}$	−141.2	5.54	5.54
$B_{12}I_{12}^{2-}$	−132.7	5.92	5.92
$B_{12}Cl_{12}^{2-}$	−147.8	5.29	5.29
$B_{12}H_{12}^{2-}$	−176.2	4.11	4.11

2. Experimental section

2.1. NMR measurements

^1H NMR and ^{11}B NMR spectra were recorded on a JEOL ECX 400 MHz NMR spectrometer.

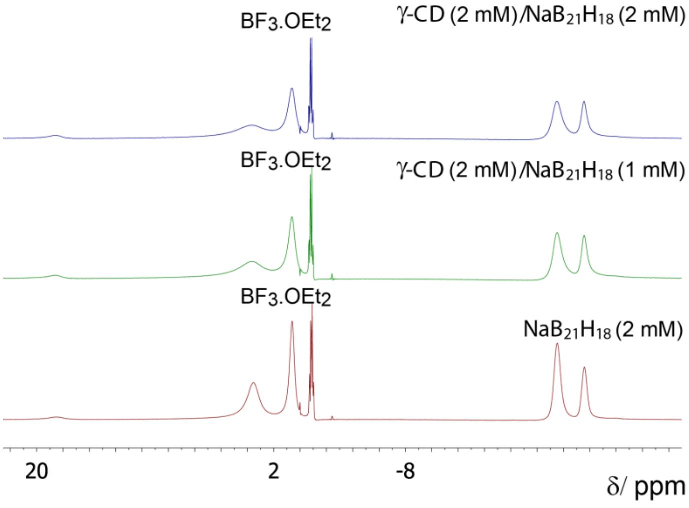

Fig. S1 ^{11}B NMR spectra of free γ-CD and the γ-CD/$NaB_{21}H_{18}$ complex, in D_2O.

2.2. ITC measurements

Isothermal titration calorimetry (ITC) experiments were carried out in water on a VP-ITC from Microcal, Inc., at 25 °C. Typically, 27 consecutive injections were used with 10 μL volume each, except for the first one which was set to 5 μL to avoid possible leakage during the equilibration time preceding the measurement. All solutions were degassed prior to titration. The first data point was removed from the data set prior to curve fitting (Origin 7.0 software) according to a one-set-of-sites model (1:1 binding stoichiometry).

2.3. Computational details. Ensembles of structures of the studied complexes were generated by MD simulations at the RI-DFT-D3/BLYP/DZVP level[1] in 1-fs steps, the Berendsen thermostat, an initial temperature of 300 K, and a target temperature of 900 K. Heavy atoms of the CD macrocycle were constrained during the simulations. Structures were collected every 0.1 ps, and the total simulation time was 1 ps. For ligands with lower symmetry, multiple simulation runs were performed with different starting geometries (three for *ortho*- and *meta*-COSAN, and two for $B_{12}H_{11}NH_3^-$). The generated structures were optimized at the RI-DFT-D3/BLYP/DZVP/COSMO level with the *lbfgs* algorithm; a change in energy of 0.006 kcal mol^{-1} and a maximal gradient of 1.2 kcal mol^{-1} $Å^{-1}$ were set as convergence criteria. Energies of the most stable structures were computed at the RI-DFT-D3/BLYP/def2-QZVP level. Hydration free energies of the free CD and of the CD complexes were computed by using the COSMO implicit solvent model[2] at the RI-DFT-D3/BLYP/def2-QZVP level, and the hydration free energies of the borate clusters were computed by using the SMD implicit solvent

model[3] at the HF/6-31G* level (the DGDZVP basis set was used for the iodine atoms). The ESPs were computed at the BLYP/def2-TZVP level.

The SMD calculations were done in Gaussian09.[4] The ESPs were computed using Gaussian09 and Molekel4.3.[5,6] The DFT-D3 calculations were performed by Cuby4 and Turbomole7.0. The D3 correction, optimization, and MD steps were done with the program Cuby4[7] which called Turbomole 7.0[8] for the DFT calculations.

3. References

(1) J. Hostaš, J. Řezáč, *J. Chem. Theory Comput.* **2017**, *13*, 3575–3585.

(2) A. Klamt, G. Schüürmann, *J. Chem. Soc., Perkin Trans* **1993**, *2*, 799–805.

(3) A. V. Marenich, C. J. Cramer, D. G. Truhlar, *J. Phys. Chem. B* **2009**, *113*, 6378–6396.

(4) M. J. Frisch, G. W. Trucks, H. B. Schlegel, G. E. Scuseria, M. A. Robb, J. R. Cheeseman, G. Scalmani, V. Barone, B. Mennucci, G. A. Petersson, H. Nakatsuji, M. Caricato, X. Li, H. P. Hratchian, A. F. Izmaylov, J. Bloino, G. Zheng, J. L. Sonnenberg, M. Hada, M. Ehara, K. Toyota, R. Fukuda, J. Hasegawa, M. Ishida, T. Nakajima, Y. Honda, O. Kitao, H. Nakai, T. Vreven, J. A. Montgomery, J. E. Peralta, F. Ogliaro, M. Bearpark, J. J. Heyd, E. Brothers, K. N. Kudin, V. N. Staroverov, R. Kobayashi, J. Normand, K. Raghavachari, A. Rendell, J. C. Burant, S. S. Iyengar, J. Tomasi, M. Cossi, N. Rega, J. M. Millam, M. Klene, J. E. Knox, J. B. Cross, V. Bakken, C. Adamo, J. Jaramillo, R. Gomperts, R. E. Stratmann, O. Yazyev, A. J. Austin, R. Cammi, C. Pomelli, J. W. Ochterski, R. L. Martin, K. Morokuma, V. G. Zakrzewski, G. A. Voth, P. Salvador, J. J. Dannenberg, S. Dapprich, A. D. Daniels, Farkas; J. B. Foresman, J. V. Ortiz, J. Cioslowski, D. J. Fox, Gaussian 09, Revision B.01. Wallingford CT, **2009**.

(5) S. Portmann, H. P. Lűthi, *Int. J. Chem.* **2007**, *54*, 766–770.

(6) P. Flűkiger, H. P. Lűthi, S. Portmann, J. Weber, Swiss Center for Scientific Computing: Manno (Switzerland), 2000.

(7) J. Řezáč, *J. Comput. Chem.* **2016**, *37*, 1230–1237.

(8) R. Ahlrichs, M. Bär, M. Häser, H. Horn, C. Kölmel, *Chem. Phys. Lett.* **1989**, *162*, 165–169.
